# Timing of cesarean and its impact on labor duration and genital tract trauma at the first subsequent vaginal birth: a retrospective cohort study

**DOI:** 10.1186/s12884-019-2359-7

**Published:** 2019-06-20

**Authors:** Zdenek Rusavy, Erika Francova, Lenka Paymova, Khaled M. Ismail, Vladimir Kalis

**Affiliations:** 1 Department of Gynecology and Obstetrics, Faculty of Medicine in Pilsen, Charles University, Prague, Czech Republic; 2 Biomedical Center, Faculty of Medicine in Pilsen, Charles University, Alej Svobody 80, 304 60 Plzen, Czech Republic; 3 Department of Obstetrics and Gynecology, Ceske Budejovice Regional Hospital, Ceske Budejovice, Czech Republic

**Keywords:** Vaginal birth after cesarean, Cervical laceration, Childbirth trauma, OASI, Perineal tear

## Abstract

**Background:**

The objectives of this study were to explore the course of labor and the risk of obstetric anal sphincter injury at the first vaginal birth after cesarean section (fVBAC) in comparison to primiparous vaginal birth (PVB) in women without epidural analgesia and to assess if laboring before the previous cesarean affected these outcomes.

**Methods:**

All fVBACs without epidural analgesia and the subsequent PVBs (controls) between 2012 and 2016 were included in this retrospective cohort study. Data were collected from health records and included maternal demographics, gestational age, and labor details (duration of 1st and 2nd stages, labor induction or augmentation, birthweight, operative vaginal birth, estimated blood loss, extent of childbirth trauma) in both groups as well as cervical dilation at the time of previous cesarean in the fVBAC group. Wilcoxon and Chi-square tests were used for data analyses.

**Results:**

The study comprised 510 women; 255 fVBACs and 255 controls. The majority of fVBACs were after a pre-labor cesarean section – 177 (69.4%). There was a statistically significant difference in the recorded duration of first stage between the fVBACs and controls (289 vs. 347 min respectively, *p* < .001). Women were less likely to have an intact perineum in the fVBAC group (29.8 vs. 43.1%, *p* < 0.01), however, there was no statistically significant difference in anal sphincter injury rates between both groups (2.3 vs. 1.9%, *p* = 0.76). The groups differed in rates of cervical tears requiring suturing (21.2 vs. 12.9%, *p* = 0.01). On further subgroup analysis, the duration of first stage of labor was shorter in women who previously had a caesarean section late in labor (≥ 8 cm cervical dilatation) compared to a pre-labor cesarean section, however, there were no differences in other outcomes.

**Conclusion:**

Compared to primiparous women having a vaginal birth, women having their first vaginal birth after a cesarean section have a shorter 1st stage of labor (particularly if the cesarean was performed in advanced labor), a higher risk of sustaining cervical lacerations and perineal trauma. However, there was no difference in the risk of sustaining obstetric anal sphincter injuries between the study groups.

**Electronic supplementary material:**

The online version of this article (10.1186/s12884-019-2359-7) contains supplementary material, which is available to authorized users.

## Background

Cesarean delivery rates are gradually increasing worldwide both in developed and undeveloped countries [1]. Reduction of Cesarean Section (CS) rates has become a priority for several health authorities globally, which might have contributed to the recently observed plateauing of this rate in some countries [2, 3]. Previous uterine scar is the most common single indication for repeat cesarean section contributing to almost a third of all cesarean deliveries in the USA [4]. Vaginal birth after cesarean section (VBAC) represents one of the effective interventions to reduce CS rates [5]. Moreover, it has been demonstrated that the use of standardized and effective protocols to inform intrapartum care and decision making in VBACs is associated with low complications and acceptable success rates [6]. However, some authors warn about the association of such practice with low success rates and relatively high risk of adverse events [7].

Although an extensively studied subject, a limited number of studies focused on pelvic floor trauma after fVBAC [8–11]. Since childbirth trauma may lead to a number of pelvic floor disorders with severe consequences, women considering a VBAC should be aware of the risk of injury during their vaginal birth. Additionally, data regarding risk of pelvic floor damage in relation to a VBAC are conflicting. While some authors reported increased obstetric anal sphincter injuries (OASIs) rates in women having their first vaginal birth after cesarean (fVBAC) [8, 10], more recent studies reported rates that are comparable to those reported in primiparous vaginal births (PVBs) [9, 11, 12] with higher rates only after previous emergency cesarean section [13]. It has been suggested that the higher risk of pelvic floor trauma is secondary to higher rates of operative vaginal birth [8].

Another possible explanation is related to the reduced cervical resistance to dilatation, and hence faster progress in labor, in parous women [14], which when coupled with a nulliparous pelvic floor may lead to higher risk of pelvic floor damage, nevertheless, this has not been properly studied [8, 15]. Therefore, the main aim of this study was to compare our primary endpoint of OASIs rate and the secondary endpoints of duration of labor and other genital tract tears in women having their fVBAC to primiparous controls who had a vaginal birth. As a secondary matter, we hypothesized that labor would be shorter and the risk of childbirth trauma would be higher in women, in whom the previous cesarean section was performed in advanced labor rather than pre-labor. Hence, we wanted to explore if laboring prior to the previous CS impacted on these outcomes.

## Methods

This is a retrospective cohort study comparing the duration of labor and genital tract trauma in women after their fVBAC compared to primiparous women who had a vaginal birth. All singleton, term (≥37th week of gestation) fVBAC who delivered at the Department of Gynecology and Obstetrics, Medical Faculty and University Hospital in Pilsen, Charles University from January 2012 till December 2016 were included in the study (Additional file 1: Figure S1). The singleton term PVB subsequent to each of the included fVBAC formed the control cohort. The controls were selected on a one-to-one ratio to the VBAC patients. Women who had a vaginal birth prior to the index cesarean section, and pregnancy complicated by intrauterine fetal death, fetal anomalies, stillbirth and those who had intrapartum epidural analgesia were excluded from the study. Since the main aim of the study was to assess the influence of the previous labor process, women with epidural analgesia were excluded as it is considered a major confounding factor for labor duration and perineal trauma [16–18]. The use of oxytocin for labor induction or augmentation or any form of pharmacologic or mechanical cervical ripening was not considered as exclusion criteria. Prostaglandins were never used for this purpose in fVBAC. The hospital clinical database was used to identify eligible women and their individual health records were used for data collection.

In our unit, the perineum is always assessed by a doctor after any type of vaginal birth and any identified trauma is classified in line with the RCOG guideline [19]. Bidigital vaginorectal examination of the anal sphincter is performed routinely in case of a suspected second degree or higher tear and episiotomy. All episiotomies were either mediolateral or lateral and cut on the woman’s right side [20] Manual perineal protection is routinely performed in our hospital for all vaginal births as previously reported [21, 22]. According to the routine practice at our institution, all women having a vaginal birth have a speculum examination immediately after the delivery of the placenta and any cervical lacerations and vaginal tears ≥5 cm in length were recorded. A vaginal tear was defined as any tear in the vaginal wall, regardless of its location or whether it was isolated or concomitant with a 1st degree perineal tear. Minimal perineal trauma was defined as non-bleeding laceration of the skin, not requiring a suture. The beginning of the first stage of labor was defined as the onset of regular contractions leading to cervical effacement or dilatation. The beginning of the second stage was defined as full dilation of the cervix. The durations of the first and the second stage of labor are recorded in minutes. Additionally, the maximum cervical dilation reached at the time of the previous cesarean section was investigated in the fVBAC group. Other institutions were asked to provide this information had the woman had her cesarean birth elsewhere. Women were excluded from the subgroup analysis if such information was not available. A subanalysis within the fVBAC group was performed based on whether the previous CS was performed pre-labor (fVBAC-PL) or in advanced labor (fVBAC-AL). This stratification was based on whether the cervix was not effaced or ≥ 8 cm at the time the decision was made to perform the CS respectively. Data were de-identified upon data collection and statistical analyses were performed using SAS 9.4 (SAS Institute Inc., Cary, NC, USA) statistical software. The comparison of variables between the two study groups with respect to their distribution of normality was performed using non-parametric ANOVA (2-sample Wilcoxon test). Categorical variables were analyzed using the χ^2^ test and described by contingency tables, *p* < 0.05 was considered statistically significant. Multivariate regression controlling for age and BMI was additionally performed for all statistically significant differences. The study was approved by the University Hospital Pilsen, Charles University ethics committee (Date of approval: 12-03-2015). Since this study was a retrospective review of electronic medical records, informed consent from the individual patients was not required.

## Results

A total of 1565 (9.7%) women with a history of CS were admitted for delivery at our referral center during the study period. A repeat CS was performed in 1189 women (76.0%) and 376 (24.0%) had a VBAC. Of these, 255 (67.8%) women were included in the study based on the *a priori* inclusion and exclusion criteria (177 (69.4%) fVBAC-PL, 31 (12.2%) fVBAC-AL) (Additional file 1: Figure S1). The control group comprised 255 PVBs who fulfilled the inclusion and exclusion criteria. The mean interval between the cesarean section in women from the VBAC group was 3.6 years.

Apart from age at the time of birth, there were no differences between the two groups in their demographic characteristics, gestational age or birthweight (Table 1). The first stage of labor was significantly shorter in the fVBAC group compared to controls (289 vs. 347 min, *p* < .001) (Table 1). A statistically significantly shorter first stage of labor in fVBAC-AL subgroup compared to fVBAC-PL (230 vs. 296 min, *p* = .007) was observed (Table 2). There were no differences in the duration of the second stage of labor, oxytocin use for labor augmentation or operative vaginal birth rates between the study groups or in the within group subanalysis, however, there were more induced labors in our control cohort (Table 1 and Table 2).

**Table 1:** Delivery characteristics

Variable	PVB (Controls)	fVBAC	*p*-value
	*n* = 255	*n* = 255	
Age [years]; mean ± SD	28.6 ± 4.8	31.7 ± 4.0	<.001^a^
BMI; mean ± SD	28.5 ± 4.8	29.1 ± 5.1	.215^a^
Gestational age [weeks]; mean ± SD	39.8 ± 1.4	39.7 ± 1.3	.107^a^
Birthweight [g]; mean ± SD	3260.6 ± 424.9	3295.6 ± 429.8	.369^a^
1st stage duration [min]; mean ± SD	347.0 ± 150.5	289.1 ± 128.1	<.001^a^
2nd stage duration [min]; mean ± SD	23.8 ± 20.6	20.8 ± 15.7	.146^a^
Instrumental delivery; n (%)	10 (3.9)	12.0 (4.7)	.663^b^
Estimated blood loss [ml]; mean ± SD	387.3 ± 185.9	391.0 ± 158.8	.039^a^
Labor induction; n (%)	34 (13.3)	3 (1.2)	<.001^b^
Oxytocin use for labor augmentation; n(%)	111 (43.5)	110 (43.1)	.929^b^

*fVBAC* First vaginal birth after cesarean

*PVB* Primiparous vaginal birth

^a^non-parametric ANOVA (2-sample Wilcoxon test)

^b^Chi-square Test

**Table 2:** fVBAC subgroups - delivery characteristics

Variable	fVBAC-PL	fVBAC-AL	*p*-value
	*n* = 177	*n* = 31	
Age [years]; mean ± SD	31.8 ± 4.1	31.9 ± 3.3	.988^a^
BMI; mean ± SD	29.5 ± 5.7	28.2 ± 3.0	.570^a^
Gestational age [weeks]; mean ± SD	39.7 ± 1.3	39.4 ± 1.3	.218^a^
Birthweight [g]; mean ± SD	3295.5 ± 408.4	3258.7 ± 269.3	.637^a^
1st stage duration [min]; mean ± SD	296.0 ± 132.7	230.0 ± 104.2	.007^a^
2nd stage duration [min]; mean ± SD	20.9 ± 15.8	22.5 ± 18.3	.806^a^
Instrumental delivery; n (%)	6 (3.4)	2 (6.5)	.340^b^
Estimated blood loss [ml]; mean ± SD	388.7 ± 166.0	433.9 ± 194.7	.077^a^
Labor induction; n (%)	1 (0.6)	1 (3.2)	.277^b^
Oxytocin use for labor augmentation; n(%)	76 (42.9)	11 (35.5)	.555^b^

*fVBAC-PL* first vaginal birth after pre-labor cesarean

*fVBAC-AL* first vaginal birth after advanced labor cesarean

a non-parametric ANOVA (2-sample Wilcoxon test)

^b^Fisher’s exact test

Women in the fVBAC group were less likely to have an intact perineum compared to PVBs (29.8 vs. 43.1%, *p* = .002), but no statistically significant difference in OASIs rates were observed (2.4% vs. 1.9%, *p* = .761) (Table 3). When comparing the fVBAC subgroups with controls for rates of 1st and 2nd degree perineal tears, the difference was only significant between fVBAC-PL and controls (15.8% vs. 9.0%, *p* = .019 and 14.7% vs. 7.8%, *p* = .022 respectively). While, the only significant difference between the two fVBAC subgroups was in episiotomy rate with it being higher in the fVBAC-AL one (61.3% vs. 38.4%, *p* = .017) (Table 4).

**Table 3:** Childbirth trauma

Variable	PVB (Controls)	fVBAC	*p*-value
	*n* = 255	*n* = 255	
Intact / minimal perineal trauma; n (%)	110 (43.1)	76 (29.8)	.002^a^
1st degree perineal rupture; n (%)	23 (9.0)	35 (13.7)	.122^a^
2nd degree perineal rupture; n (%)	20 (7.8)	33 (12.9)	.059^a^
3rd degree perineal rupture; n (%)	5 (1.9)	6 (2.4)	.761^a^
Episiotomy; n (%)	102 (40.0)	109 (42.8)	.579^a^
Important vaginal tear ≥5 cm; n (%)	32 (12.5)	45 (17.3)	.136^a^
Cervical laceration ≥1 cm; n (%)	33 (12.9)	54 (21.2)	.014^a^

*fVBAC* First vaginal birth after cesarean

*PVB* Primiparous vaginal birth

^a^Chi-square Test

**Table 4:** fVBAC subgroups - childbirth trauma

Variable	fVBAC-PL	fVBAC-AL	*p*-value
	*n* = 177	*n* = 31	
Intact / minimal perineal trauma; n (%)	53 (29.9)	5 (16.1)	.132^b^
1st degree perineal rupture; n (%)	28 (15.8)	3 (9.7)	.584^b^
2nd degree perineal rupture; n (%)	26 (14.7)	4 (13.0)	1.00^b^
3rd degree perineal rupture; n (%)	4 (2.3)	1 (3.2)	.558^b^
Episiotomy; n (%)	68 (38.4)	19 (61.3)	.017^a^
Important vaginal tear ≥5 cm; n (%)	32 (18.1)	4 (13.0)	.612^b^
Cervical laceration ≥1 cm; n (%)	38 (21.5)	3 (9.7)	.149^b^

*fVBAC-PL* first vaginal birth after pre-labor cesarean

*fVBAC-AL* first vaginal birth after advanced labor cesarean

^a^Chi-square Test

^b^Fisher’s exact test

There were no statistically significant differences in rates of vaginal tears in any of the between or within group analysis, however, cervical lacerations ≥1 cm were more frequent in the fVBAC group compared to controls (21.2% vs. 13.0%, *p* = .014). When comparing fVBAC subgroups with controls, a similar pattern was only observed between the fVBAC-PL vs control but not the fVBAC-AL vs control subanalysis (21.5% vs. 13.0%, *p* = .027 and 9.7% vs. 13.0%, *p* = .779 respectively). Nonetheless, the difference between cervical tears between the two fVBAC subgroups did not reach statistical significance, which could be a reflection of the relatively small sample size of the fVBAC-AL subgroup. All observed differences remained statistically significant on multivariate analysis controlling for maternal age and BMI.

## Discussion

The findings of this study demonstrate that women having their fVBAC are not similar to PVBs with regards to several labor and birth outcomes. Indeed, our hypothesis, that women having the fVBAC would have a shorter first stage of labor compared to PVBs was confirmed. Furthermore, this difference was more striking in women who were advanced in labor prior to having their CS. In spite of identifying a higher risk of spontaneous perineal tears and episiotomy in general in the fVBAC cohort compared to controls, there were no differences in OASIs between the two groups. Finally, on subgroup analysis the risk of cervical laceration was almost doubled in the subgroup of fVBACs who did not labor before compared to PVBs. Contrary to previous studies, we did not find increased OASIs rate [8, 10]. It is possible that the reason for this is that, unlike other studies, the birthweight and operative vaginal birth rates, which are known risk factors for OASIs, were comparable between our two study groups [8, 15]. Another possible reason for the low OASIs rate could be related to the strong research focus in our department on reducing the risk childbirth-related pelvic floor trauma and the staff receive regular training related to intrapartum interventions for the primary prevention of OASIs. The method of assessment of perineal trauma is not fully described in most articles that have suggested a higher OASIs rate following VBAC and it is possible that after VBAC women were subject to a more systematic assessment or the examination was performed by a more experienced accoucher and either could have enhanced the detection rate [23]. In our unit, the perineum is always inspected using the recommended bidigital vaginorectal examination by an experienced obstetrician or midwife [19]. Finally, it is important to highlight that operative vaginal birth rates are generally very low in the Czech Republic with a preference towards the use of ventouse because of its associated lower risk of OASIs compared to forceps [24].

Intrapartum cervical lacerations are relatively common with an overall incidence widely ranging from 25 to 90% [25], most of these are detectable only on routine cervical examination after a vaginal birth. Although a routine policy in our unit, this practice is not adopted in the majority of maternity units globally either because it is considered an uncomfortable intervention for the women or because of the perceived lack of association between small cervical laceration and poor outcomes. We appreciate that the cervical laceration rates described in the present study are high in comparison to previously published data [25–27], however, this might be a reflection of the severity of reported lacerations where other studies focused on more severe cervical lacerations that were associated with severe postpartum hemorrhage or involvement of other structures like the lower uterine segment or the vaginal wall [25]. Cervical lacerations of 1 cm and more are considered clinically significant and sutured at our institution. This threshold was therefore selected for comparison. Investigating the reason for the observed high cervical tear rates was beyond the scope of our study and hence our proposed reasoning for this finding is only speculative. It is plausible that the higher risk of cervical laceration in the VBAC group could be linked to non uniform reduction in cervical tissue resistance up to the degree to which the cervix has previously dilated causing a mismatch between the strength of uterine contractions and cervical resistance resulting in its traumatization. However, this explanation does not support the finding that on subgroup analysis the difference in laceration was only significant when the PVB versus fVBAC-PL comparison.

Our results regarding the difference in labor duration are in contradiction with previously published studies. A secondary analysis of data from a Consortium on Safe Labor [4] study has shown that labor duration for a trial of labor after cesarean was slower compared to nulliparous labor [28]. Other studies described comparable first stage and shorter second stage of labor duration in VBAC [12, 15]. We do not have a clear explanation for these inconsistencies and we can only speculate that the difference could be a result of the exclusion of women who had epidural analgesia or the relatively low operative vaginal birth rates in our study. We identified a higher episiotomy rate in our fVBAC-AL compared to fVBAC-PL group. There is no clear explanation of this observation because the majority of episiotomies are performed at the accoucher’s discretion and the indication for the episiotomy is not routinely documented. On detailed review of the hospital notes of women who had an episiotomy in both subgroups, 21/68 episiotomies in fVBAC PL group and 5/19 episiotomies in the fVBAC-AL had evidence of suspected fetal distress, which might have been the indication for the episiotomy, nevertheless this does not explain the findings. It is possible that this difference is a reflection of a faster progressing labor and “nulliparous” perineum. Interestingly, a recent study reported an increased risk of OASIs in women delivering vaginally after emergency compared to elective caesarean sections and episiotomy appeared to be protective [13]. The increased rate of episiotomy in our fVBAC-AL group might explain the comparable OASIs rates in our study.

The major strength of the present study lies in its design. Unlike most previous studies on this topic, this is not a registry analysis. Hand abstracting of the results allowed for looking at individual health records to obtain more precise and detailed data. Additionally, since all women had their fVBAC in the same institution, the variation in obstetric practice and perineal management during labor would have been minimal. Another strength of the study is that the study groups were well defined where we did not include any women with previous vaginal deliveries or those who used epidural analgesia, both are established confounders to the outcomes of interest. Moreover, we were able to perform some preliminary analyses on the impact of the type of CS performed on the course of subsequent VBAC. In this context, we intentionally only included women who had a planned CS with a non-effaced cervix and those who had an emergency CS after being advanced in labor because we hypothesized that if there was a difference in any of these outcomes it will be more evident between these two distinct clinical categories. The major limitation of the study is certainly the number of women in the fVBAC-AL group, however, the size still allowed a proper statistical analysis. Another limitation is the absence of ultrasound assessment in the follow-up. The assessment of the perineal trauma was performed clinically after the delivery. It was suggested that more than a half of OASIs may remain undiagnosed by the attending obstetrician or midwife, providing rectal examination is not performed after the delivery [23]. However, this examination is part of our routine practice. Nonetheless, in this retrospective analysis we were unable to objectively assess and quantify anal sphincter and levator ani injuries using ultrasound in follow-up. This remains an objective for future studies. Finally, although the exclusion of women who used an epidural allowed a more robust evaluation of our hypotheses, it limits extrapolating our study findings to women opting to use epidural analgesia for their fVBAC.

## Conclusions

In conclusion, compared to primiparous women having a vaginal birth, women having their first vaginal birth after a cesarean section without epidural analgesia have a shorter 1st stage of labor. This difference is more pronounced if the woman’s previous cesarean was performed in advanced labour. Women having their fVBAC seem to have a higher risk of sustaining cervical lacerations and perineal trauma. However, the risk of anal sphincter injuries does not seem to be increased which is reassuring for women considering a trial of VBAC.

## Additional file

Additional file 1: **Figure S1.** Identification of fVBAC study participants. (DOCX 26 kb)

## Data Availability

The datasets generated and/or analyzed during the current study are available in the Figshare online open access repository, 10.6084/m9.figshare.7732898

## Abbreviations

CS: cesarean section
fVBAC: first vaginal birth after cesarean
fVBAC-AL: first vaginal birth after advanced labor cesarean
fVBAC-PL: first vaginal birth after pre-labor cesarean
OASI: obstetric anal sphincter injury
PVB: primiparous vaginal birth
VBAC: vaginal birth after cesarean
