# Supplementary material for: Timing of cesarean and its impact on labor duration and genital tract trauma at the first subsequent vaginal birth: a retrospective cohort study
Source: BMC Pregnancy Childbirth. 2019 Jun 20;19:207. doi: 10.1186/s12884-019-2359-7 (PMC6585007; doi:10.1186/s12884-019-2359-7)
Supplement: Supplementary file 1 — Figure S1. Identification of fVBAC study participants. (DOCX 26 kb) [file 12884_2019_2359_MOESM1_ESM.docx]

**Additional file 1: Figure S1: Identification of fVBAC study participants**
